# Use of the Guardian table extension during periacetabular osteotomy is associated with an increased risk of intraoperative posterior column fractures

**DOI:** 10.1093/jhps/hnaf062

**Published:** 2025-10-17

**Authors:** Ta-Wei Tai, Emil Varas-Rodríguez, Emmanouil Grigoriou, Sergio F Guarin Perez, Diego J Restrepo, Rafael J Sierra

**Affiliations:** Department of Orthopedics, National Cheng Kung University Hospital, College of Medicine, National Cheng Kung University, 138, Shengli Rd., North Dist., Tainan City 704, Taiwan; Department of Orthopedic Surgery, Mayo Clinic, 200 First St. SW Rochester, MN 55905, United States; Department of Orthopedic Surgery, Mayo Clinic, 200 First St. SW Rochester, MN 55905, United States; Department of Orthopedic Surgery, Mayo Clinic, 200 First St. SW Rochester, MN 55905, United States; Department of Orthopedic Surgery, Mayo Clinic, 200 First St. SW Rochester, MN 55905, United States; Department of Orthopedic Surgery, Mayo Clinic, 200 First St. SW Rochester, MN 55905, United States; Department of Orthopedic Surgery, Mayo Clinic, 200 First St. SW Rochester, MN 55905, United States

**Keywords:** periacetabular osteotomy, Guardian table, posterior column fracture, hip arthroscopy, surgical complications

## Abstract

The Guardian table is widely utilized for combined hip arthroscopy and periacetabular osteotomy (PAO) due to its postless traction design and the ability to perform both procedures without the need for redraping. This retrospective cohort study reports one surgeon’s use of the Guardian table extension during combined hip arthroscopy and PAO and the occurrence of intraoperative posterior column fractures. Seventy-two consecutive PAO procedures performed at a single institution between February 2023 and January 2025 were analysed. Procedures involving concomitant hip arthroscopy (*n* = 26) utilized the Guardian table, whereas those without arthroscopy (*n* = 46) used a conventional radiolucent table. Patient demographics, clinical data, and complications were reviewed. Additionally, mechanical testing quantified table stability by measuring peak absolute acceleration (‘table bounce’) using accelerometry during simulated malleting. The incidence of intraoperative posterior column fractures was significantly higher with the Guardian table (4/26, 15.4%) compared to the conventional table (0/46, 0%; *P* = .0062). Mechanical testing demonstrated significantly greater table bounce for the Guardian table (69.1 ± 3.4 m/s^2^ versus 28.2 ± 4.4 m/s^2^, *P* < .001). Patient characteristics and surgical complexity did not differ significantly between fracture and non-fracture cases. These findings suggested that the Guardian table extension system was associated with increased risk of posterior column fractures, likely due to lack of distal support and stability. Surgeons should consider alternative table setups or intraoperative support modifications to mitigate this risk during PAO procedures.

## INTRODUCTION

Periacetabular osteotomy (PAO) is a well-established surgical procedure for treating symptomatic developmental dysplasia of the hip (DDH) [1, 2]. The procedure involves controlled osteotomies around the acetabulum, allowing for reorientation to improve femoral head coverage and joint stability. Successful execution of PAO requires precise osteotomy and fragment manipulation, which depend heavily on stable patient positioning and reliable intraoperative imaging.

Patients undergoing PAO frequently present with concomitant intraarticular pathology, such as labral tears, chondral damage, or femoroacetabular impingement. As a result, combined hip arthroscopy and PAO have become increasingly popular, although routinely adding arthroscopy remains debated [3].

In recent years, the Guardian table (Stryker, Greenwood Village, CO) has gained popularity for hip surgeries, particularly for hip arthroscopy. This table is specifically designed to optimize patient positioning and traction during arthroscopic procedures [4]. It allows postless traction to prevent complications related to the perineal posts [5, 6]. Once completed the table then allows to proceed with the PAO without redraping.

The suitability of the Guardian table for bony procedures such as PAO has not been thoroughly evaluated. The extension attachment, composed of a carbon fibre rod and foam pad, has a flexible structure without any distal support. This flexibility may compromise table stability during bone work, especially during high-force manoeuvres such as malleting the Ganz or Mast osteotomes for ischial and posterior column osteotomies. This is particularly important during the ischial osteotomy as the mallet force is driven posteriorly. The lack of support and recoil intraoperatively seemed to require additional malleting to achieve the same results into the bone. This may transmit unintended stress to the posteriorcolumn, potentially increasing the risk of intraoperative fracture—an uncommon but serious complication [7].

To investigate this potential issue, we analysed clinical data from our recent PAO cases performed after the introduction of the Guardian table at our institution. The primary aim of this study was to evaluate whether use of the Guardian table during combined hip arthroscopy and PAO was associated with an increased risk of intraoperative posterior column fractures compared to PAO performed on a conventional radiolucent table. Secondary aims included identifying potential risk factors for intraoperative fractures by comparing patients in the Guardian table group with and without such complications. As a final exploratory aim, we performed a mechanical assessment using accelerometry to compare table stability—measured by peak absolute acceleration (‘table bounce’)—across different table setups, in order to investigate a possible biomechanical mechanism underlying the increased fracture risk.

## METHOD

### Patient population

This retrospective cohort study included all patients undergoing PAO at our institution between February 2023 and January 2025, with a secondary, exploratory biomechanical assessment of table stability. During this 2-year period, PAOs were performed on the Guardian table if concomitant arthroscopy was planned, while PAOs without arthroscopy were performed on a regular radiolucent operating table. This study was approved by the institutional review board, and all procedures were conducted in accordance with institutional and ethical guidelines.

A total of 72 consecutive PAO cases performed at our institution were reviewed. The indication for concomitant hip arthroscopy included preoperative physical examination findings and MRI evidence of intraarticular lesions, such as labral tears, femoroacetabular impingement, or cartilage damage. Among these patients, 26 procedures (36.1%) were performed with concomitant arthroscopy on the Guardian table system, and the remaining 46 cases were performed on a regular radiolucent operating table (Skytron Elite 3 000, Skytron, Grand Rapids, MI, USA). Patient-level data collected included age, sex, body mass index (BMI), smoking status, diagnosis, and ASA physical status. Bone mineral density testing was not routinely performed, as the PAO population consisted predominantly of young patients without clinical risk factors for osteoporosis. Intraoperative variables included estimated blood loss, skin-to-skin operation time, and whether concomitant hip arthroscopy was performed. Postoperative complications were recorded, including intraoperative posterior column fracture, femoral nerve palsy, lateral femoral cutaneous nerve (LFCN) dysesthesia, symptomatic heterotopic ossification (HO), superficial wound infection, and need for reoperation. There were 13 male and 59 female patients. The mean age of the enrolled patients was 23.1 ± 7.4 years [mean ± standard deviation (SD)], and the mean BMI was 24.2 ± 3.8. There were no significant differences between patients undergoing PAO on the Guardian table and those on the regular operating table in terms of age, BMI, diagnosis, or smoking history.

### Surgical technique and concomitant procedures

All PAO procedures were performed by a senior orthopaedic surgeon (R.J.S.) experienced in hip preservation surgery. For cases with concomitant hip arthroscopy, the arthroscopy was performed by the same surgeon first, using the Guardian table with leg holders and a traction system in the supine position. Arthroscopic examination, debridement, and labral repair with suture anchors were performed based on the grade of labral lesions. Among the 26 patients undergoing arthroscopy in this cohort, 17 underwent labral repair, 7 underwent acetabular chondroplasty, and 9 underwent head/neck junction osteochondroplasty.

After completing the arthroscopy, the portal stab wounds were closed, and the leg holders and traction system were unclamped, and the Guardian table leg extension attachment was assembled to the table to hold the lower limbs. Then, the PAO was subsequently completed. For cases without arthroscopy, PAO was performed directly on a regular radiolucent operating table in the supine position.

### PAO surgical technique

Following patient repositioning and re-draping, a slightly curved longitudinal incision centred over the anterior superior iliac spine (ASIS) was made. The Smith-Petersen approach was utilized, with care taken to preserve the abductor and quadriceps femoris muscles. The osteotomies were performed under fluoroscopic guidance. The ischial osteotomy was performed using a Ganz osteotome and mallet, advancing to approximately half the thickness of the ischium. The iliac osteotomy was performed using an oscillating saw. The posterior column osteotomy was completed using a mallet and various curved osteotomes to connect anteriorly with the ischial cut, fully freeing the acetabular fragment. The acetabular fragment was then mobilized into the desired position using a Schanz screw and bone hook under fluoroscopic control. The fragment was fixed using three cortical screws. If an intraoperative posterior column fracture was identified on fluoroscopy, it was stabilized with an additional screw if necessary.

### Postoperative care and follow-up

Patients were observed overnight and began ambulation with crutches using touch weight bearing for the first month, followed by partial weight bearing for 1 month, and gradual progression to full weight bearing thereafter. Follow-up visits were scheduled at 2 months, 4 months, and 1 year postoperatively. For patients with intraoperative posterior column fractures, the rehabilitation programme was delayed by 2–4 weeks, depending on the healing status of the osteotomy and fracture sites.

### Table bounce measurement

A standardized mechanical evaluation was conducted to quantify table bounce. An iPhone 15 Pro Max, equipped with the Phyphox application (RWTH Aachen University), was securely mounted at the distal end of the operating table to function as an accelerometer. A 5-kg sandbag was released from a vertical height of 10 cm onto the table surface, positioned 100 cm proximal to the device. The impact was applied at 2-s intervals for a total of 20 repetitions. Prior to the formal data collection, five pilot tests were conducted to refine and standardize the protocol. The final experimental procedure was designed to reduce measurement variability, achieving an SD of <20% across repeated trials. While not intended as a validated biomechanical construct test, this method was sufficient to illustrate relative differences in table compliance, supporting our clinical observations. This procedure was performed under three conditions: (i) a conventional radiolucent operating table, (ii) the Guardian table with a flat extension attachment, and (iii) the Guardian table with a height-adjustable stool positioned underneath and directly supporting the distal table end to enhance stability (Fig. 1). For each setting, the Phyphox app recorded acceleration data, and the mean peak absolute acceleration was calculated. This provided a quantitative comparison of table bounce across different configurations.

**Figure 1:**
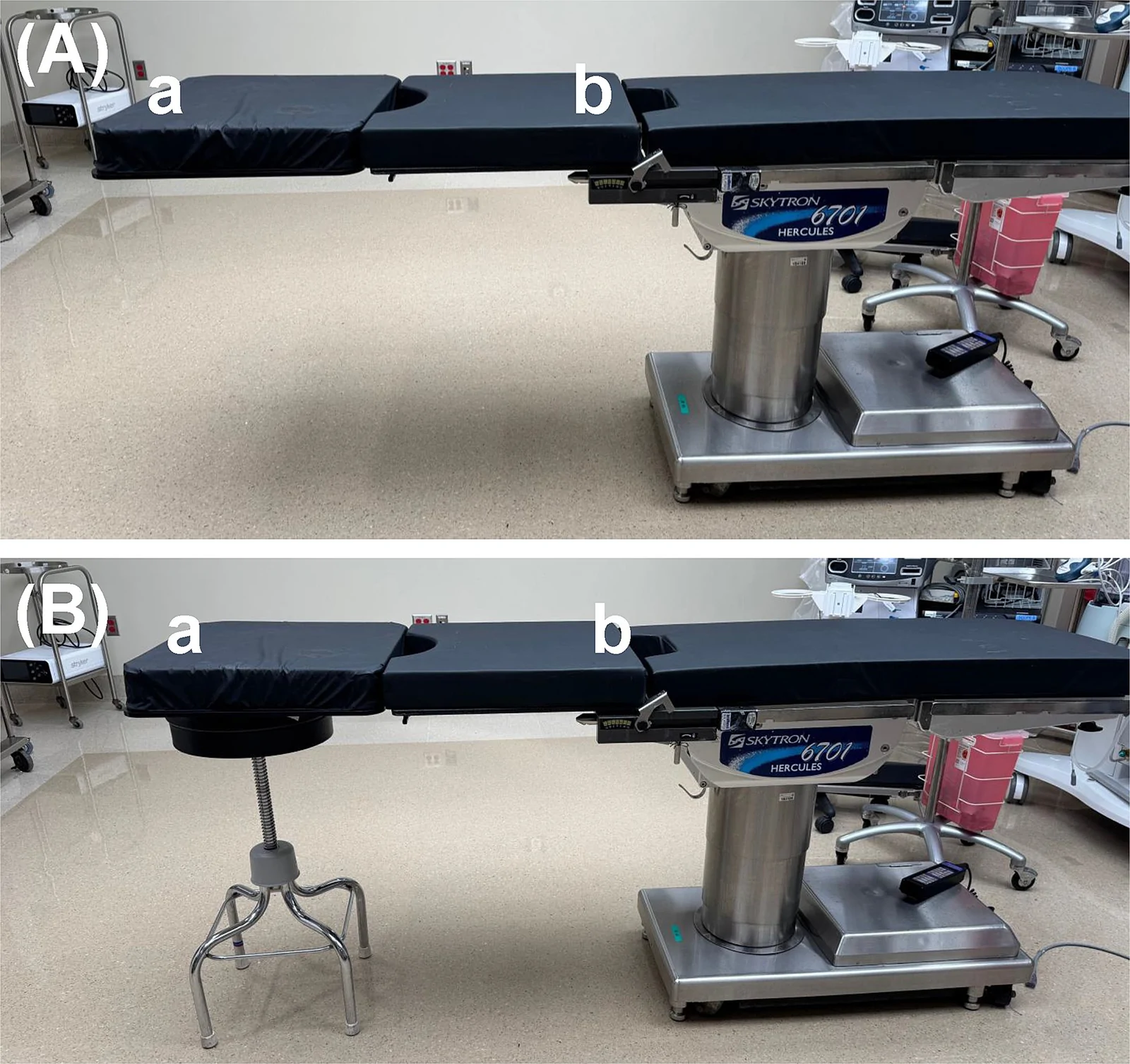
Experimental configurations for assessing table bounce. (A) Guardian table flat leg extension attachment alone. (B) Guardian table flat leg extension attachment with a height-adjustable stool placed beneath and in direct contact with the distal end of the extension to provide additional support. For each setup, an iPhone 15 Pro Max running the Phyphox application was securely attached at the table’s distal tip (point a) to record acceleration data. A 5-kg sandbag was dropped from 10 cm height onto the tabletop at a point located 100 cm from the accelerometer (point b), repeated 20 times at 2-s intervals.

### Data analysis

The incidence of intraoperative posterior column fractures was compared between the two table groups using Fisher’s exact test. Baseline patient characteristics were compared using Student’s *t*-test for continuous variables and Fisher’s exact test for categorical variables. Table bounce across the three configurations was compared using one-way analysis of variance (ANOVA), followed by pairwise post-hoc comparisons with Holm-adjusted *P*-values. A *P*-value of < .05 was considered statistically significant. All statistical analyses were performed using BlueSky Statistics software version 10.3.4 (Chicago, IL, USA).

## RESULTS

### Clinical data

The incidence of intraoperative posterior column fractures was significantly higher in the Guardian Table group compared to the conventional table group (4/26, 15.4% versus 0/46, 0%; *P* = .0062) (Table 1). The operation time was significantly longer in the Guardian Table group (170 ± 54 min versus 90 ± 18 min, *P* < .0001), which was expected since all patients in this group underwent concomitant hip arthroscopy prior to the PAO. Additionally, patients in the Guardian group had a significantly higher proportion of female patients (96.2% versus 73.9%, *P* = .0184), more frequent use of general anaesthesia (73.1% versus 41.3%, *P* = .0095), and a lower proportion of ASA status ≥2 (26.9% versus 52.2%, *P* = .0377). There were no significant differences in age, BMI, smoking history, or estimated blood loss between the two groups.

**Table 1:** Basic characteristics of patients undergoing PAO using the Guardian table extension or a conventional radiolucent table.

**Characteristics**	**PAO on Guardian Table** **(*n* = 26)**	**PAO on** **Conventional Table** **(*n* = 46)**	***P* value**
**Age**	24.2 ± 8.6 range 14–45	22.5 ± 6.7 range 16–45	.3954
**Gender** Women Men	25 (96.2) 1 (3.8)	34 (73.9) 12 (26.1)	.0184^*^
**BMI**	23.3 ± 2.9	24.8 ± 4.2	.0725
Overweight (BMI ≥ 25)	7 (26.9)	20 (43.5)	.1634
Obesity (BMI ≥ 30)	2 (7.7)	8 (17.4)	.2530
**Smoking history**	4 (15.4)	6 (13.0)	.7826
**Previous hip arthroscopy**	2 (7.7)	12 (26.1)	.0582
**Diagnosis**			
Dysplasia	25 (96.2)	46 (100)	.1804
Retroversion	1 (3.8)	1 (2.2)	.6783
**ASA status** ≥ 2	7 (26.9)	24 (52.2)	.0377^a^
**General anaesthesia**	19 (73.1)	19 (41.3)	.0095^a^
**Concomitant arthroscopy**	26 (100)	0 (0)	<.0001^a^
**Estimated blood loss (ml)**	624 ± 401	506 ± 256	.1341
**Operation time (minutes)**	170 ± 54	90 ± 18	<.0001^a^
**Intraoperative posterior column fracture**	4 (15.4)	0 (0)	.0062^a^

BMI, body mass index (kg/m^2^). ASA status, American Society of Anaesthesiologists physical status classification. Age, BMI, estimated blood loss, and operation time are presented as the means ± SDs, while other values are shown as numbers (%). A *P*-value < .05 indicates a significant difference between the groups with and without complications.

To further investigate potential risk factors contributing to intraoperative posterior column fractures, we compared characteristics between patients using the Guardian Table with and without such fractures (Table 2). No significant differences were found in terms of age, gender, BMI, ASA status, type of arthroscopic procedure, blood loss, or operation time.

**Table 2:** Characteristics of patients undergoing PAO using the Guardian table extension with or without intraoperative posterior column fracture.

**Characteristics**	**With Intraoperative posterior column fracture (*n* = 4)**	**Without Intraoperative posterior column fracture (*n* = 22)**	***P* value**
**Age**	25.3 ± 10.2 range 17–40	24.0 ± 8.5 range 14–45	.8239
**Gender** Women Men	4 (100) 0 (0)	21 (95.5) 1 (4.5)	.6637
**BMI**	24.1 ± 2.1	23.1 ± 3.0	.4406
Overweight (BMI ≥ 25)	2 (50.0)	5 (22.7)	.2580
Obesity (BMI ≥ 30)	0 (0)	2 (9.1)	.5302
**Diagnosis**			
Dysplasia	4 (100)	21 (95.5)	.6637
Retroversion	0 (0)	1 (4.5)	.6637
**Smoking**	1 (25.0)	3 (13.6)	.5623
**ASA status** ≥ 2	1 (25.0)	6 (27.3)	.9249
**General anaesthesia**	3 (75.0)	16 (72.7)	.9249
**Arthroscopic procedure**			
Labral repair	3 (75.0)	13 (59.1)	.5474
Head–neck junction osteochondroplasty	1 (25.0)	7 (31.8)	.7858
Acetabular chondroplasty	0 (0)	6 (27.3)	.2337
**Blood loss**	588 ± 440	491 ± 221	.6939
**Operation time (minutes)**	146 ± 30	174 ± 57	.1841

BMI, body mass index (kg/m^2^). ASA status, American Society of Anaesthesiologists physical status classification. Age, BMI, and operation time are presented as the means ± SDs, while other values are shown as numbers (%). A *P*-value < .05 indicates a significant difference between the groups with and without complications.

Other complications were infrequent and similar in both groups. In the Guardian Table group, complications included one case each of femoral nerve palsy, LCFN dysesthesia, symptomatic HO, and superficial wound infection treated with oral antibiotics. In the conventional table group, one case of a delayed healing wound was reported.

Among the four posterior column fractures identified in the Guardian table group, three were detected on immediate postoperative radiographs, while one was recognized intraoperatively under fluoroscopic guidance. The fracture line typically originated at the junction between the ischial and posterior column osteotomies and extended posteriorly through the posterior column. All of them are female. One case involved a 40-year-old woman with an initially unrecognized fracture who reported persistent ischial pain during recovery. Follow-up imaging at 7 months revealed a nonunion. Although the patient had undergone screw fixation, the screw failed within 2 months, necessitating revision with plate fixation. Despite this intervention, union was not achieved at 6-month follow-up after plate fixation (Fig. 2). Another case, detected intraoperatively, was stabilized with a single anterior-to-posterior screw and progressed to uneventful healing (Fig. 3). The remaining two cases were identified on postoperative radiographs; these patients were prescribed an additional four weeks of non-weight-bearing ambulation. Both demonstrated satisfactory healing on follow-up imaging without further complications.

**Figure 2:**
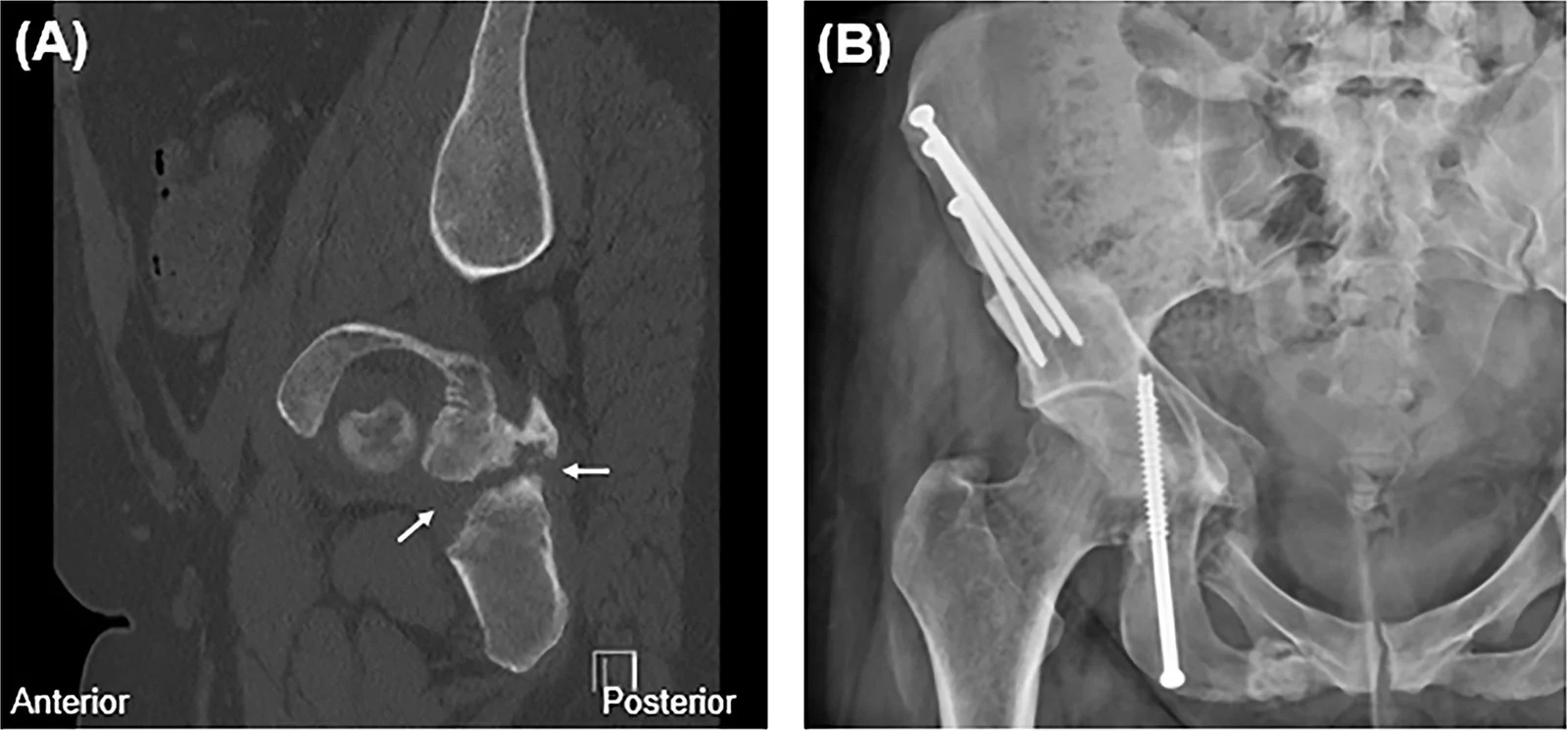
A 40-year-old woman underwent right PAO and labral repair, complicated by posterior column fracture. Sagittal CT at 7 months postoperatively shows a nonunion of the posterior column (arrow) (A). Due to persistent pain, percutaneous retrograde screw fixation from the ischium was performed for stabilization (B).

**Figure 3:**
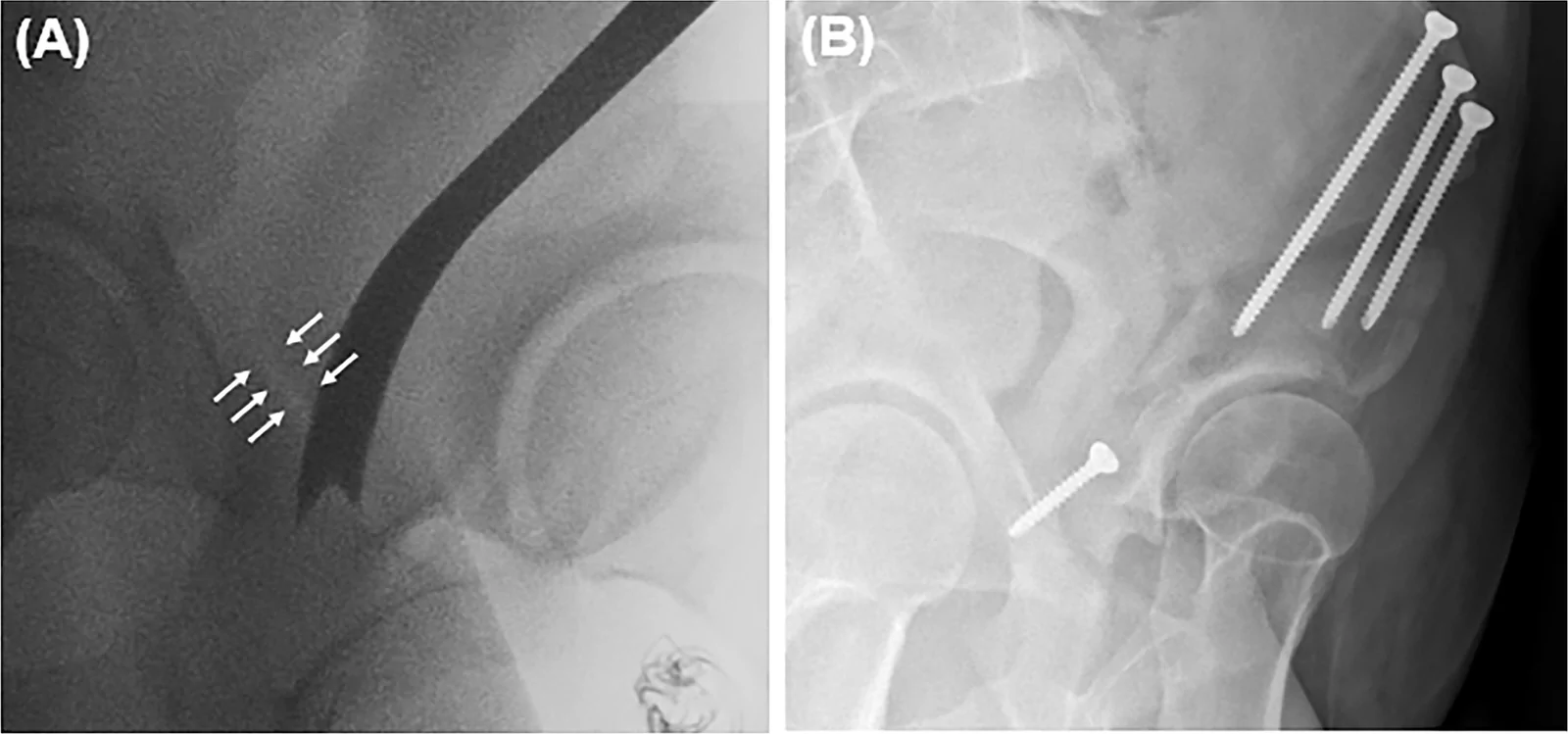
A 21-year-old woman underwent left hip arthroscopy followed by PAO. Intraoperative fluoroscopy with a 60° oblique view revealed propagation of the ischial osteotomy into the posterior column (arrow) (A). An additional 4.5 mm, 30 mm screw was placed for fixation. False profile lateral standing view radiograph at 8-week follow-up showed healing without further displacement (B).

### Table bounce

The simulated PAO impact testing demonstrated a statistically significant difference in table bounce among the three configurations (*P* < .001, one-way ANOVA). The Guardian table with flat extension attachment showed the highest mean peak absolute acceleration (69.1 ± 3.4 m/s^2^), followed by the same table supported distally with a stool (35.2 ± 3.2 m/s^2^), and the conventional radiolucent table (28.2 ± 4.4 m/s^2^). Pairwise post-hoc analysis confirmed significant differences between all groups (all *P* < .001). The addition of stool support to the Guardian table substantially reduced vibration but remained significantly higher than the conventional table (*P* < .001). (Fig. 4).

**Figure 4:**
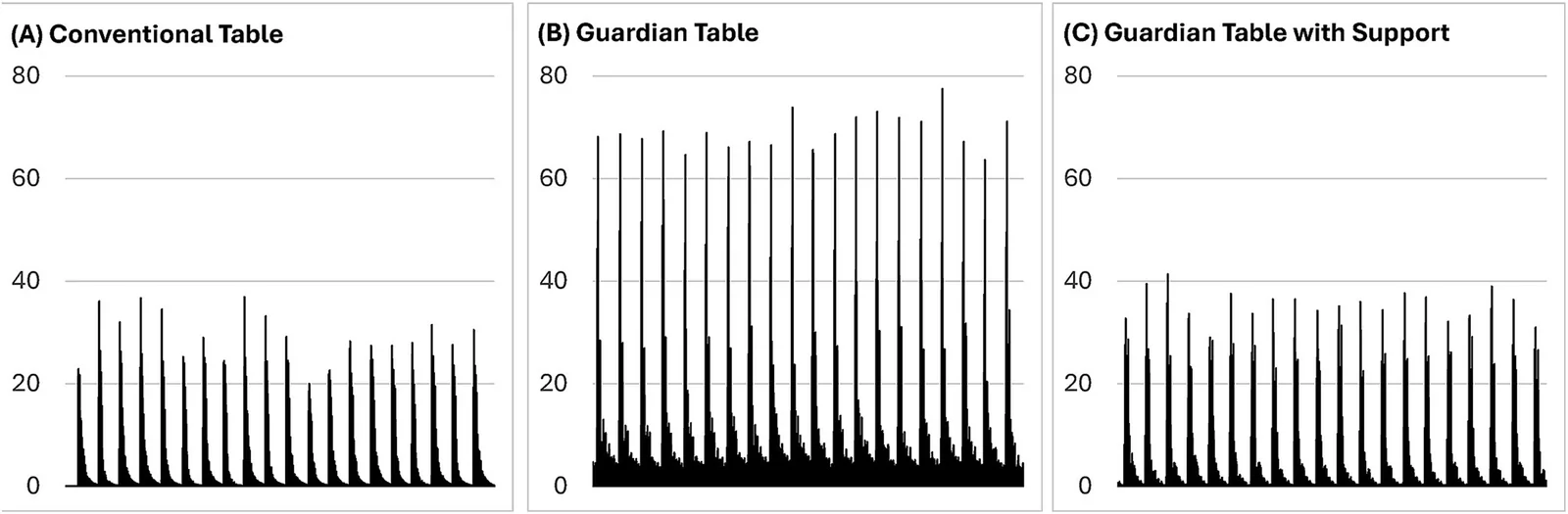
Absolute acceleration (m/s^2^) recordings during simulated impact testing on the conventional radiolucent table (A), the Guardian table with flat extension attachment (B), and the Guardian table with additional distal support (C). The Guardian table demonstrated the highest mean peak absolute acceleration, which was significantly greater than both the conventional table and the Guardian table with support.

## DISCUSSION

Our study demonstrated a significantly increased risk of intraoperative posterior column fractures when performing PAO on the Guardian table using the flat extension attachment. Within the Guardian table group, patient characteristics, surgical complexity, and arthroscopic procedures were not significantly different between cases with and without intraoperative posterior column fracture. Despite this, the overall incidence of fracture was significantly higher in the Guardian group compared to procedures performed on the conventional radiolucent table, suggesting that table setup may be the primary contributing factor. Objective mechanical testing further supported this finding, revealing greater peak absolute acceleration (i.e. table bounce) with the Guardian setup during simulated malleting. Together, these clinical and biomechanical data suggest that the increased compliance of the guardian bed extension may be a contributing factor predisposing to posterior column fractures.

Posterior column fractures occurring during or after PAO, while relatively rare, represent significant complications. Postoperative stress fractures are reported more frequently, with some series noting incidences as high as 15% [7]. In contrast, intraoperative posterior column fractures occur in fewer than 5% of cases according to existing literature [8]. Despite their low incidence, such fractures can critically undermine the posterior column’s structural integrity, leading to acetabular instability, nonunion, chronic pain, and suboptimal clinical outcomes [7–9].

Preventing intraoperative posterior column fractures during PAO requires a thorough understanding of pelvic anatomy and the application of precise surgical techniques. A critical step is to carefully monitor the trajectory and insertion depth of the osteotomes with fluoroscopic assistance [7, 8, 10]. During the ischial osteotomy, the instrument should remain centred and should not penetrate beyond the midpoint of the ischium, as deeper advancement increases the risk of damaging the posterior column. To safely connect the retroacetabular and ischial cuts, a straight osteotome is used initially, followed by a transition to a curved osteotome once the trajectory begins to diverge from the subchondral surface. This curved approach minimizes stress on the surrounding bony structures and reduces the chance of unintended fracture propagation.

While minimally displaced fractures may be managed conservatively with restricted weight-bearing, unstable fractures typically necessitate surgical stabilization with screws or plates [7, 11]. In this series, four intraoperative posterior column fractures were documented. One patient underwent immediate screw fixation and recovered without delay. Of the three who did not receive fixation, two recovered uneventfully, while one developed persistent ischial pain during ambulation that eventually required secondary fixation. If recognized intraoperatively, additional fixation is recommended [12].

The Guardian table facilitates seamless integration of hip arthroscopy with PAO, eliminating the need to transfer patients between tables. Its postless design may reduce the risk of complications associated with the use of a perineal post during traction in hip arthroscopy [4–6]. To enhance stability of the flat extension, some surgeons have introduced a height-adjustable chair positioned beneath the extension plate to provide additional support. Preliminary observations suggest that this simple mechanical support may improve construct rigidity but is not equivalent to a standard image table. Further investigation is warranted to determine whether increased stiffness translates to a lower risk of intraoperative fractures.

There are several limitations to this study. First, the retrospective nature and non-randomized table assignment may introduce selection bias, as the Guardian table was used only in cases requiring concomitant arthroscopy. This inherent association limits the ability to completely isolate table type as the sole contributor to fracture risk. Second, our sample size, particularly within the Guardian subgroup with fractures, was relatively small, which may reduce the power to detect smaller differences in associated risk factors. Third, while the accelerometer simulation provides quantitative insight into table bounce, it does not perfectly replicate *in vivo* surgical dynamics. Nevertheless, this simple test reflects the real-world concern raised by experienced surgeons during PAO procedures.

In conclusion, the use of the Guardian table extension system during PAO, especially following concomitant arthroscopy, may be associated with an increased risk of intraoperative posterior column fractures. This risk appears to be related to mechanical instability stemming from the flexible flat extension attachment. Until further modifications are implemented or alternative solutions are developed, surgeons should be aware of this potential complication when utilizing this setup and consider augmenting intraoperative support or selecting more rigid configurations when posterior column osteotomy is performed.

## Data Availability

The data that support the findings of this study are available from the corresponding author upon reasonable request.
